# Dr. Wayne Feng, Professor of Biomedical Engineering, Pratt School of Engineering, Duke University, United States: Adapted Interview from the 12^th^ World Congress for NeuroRehabilitation (WCNR), Vienna, 2022

**DOI:** 10.25122/jml-2023-1016

**Published:** 2023-04

**Authors:** Stefana-Andrada Dobran, Alexandra Gherman

**Affiliations:** 1 RoNeuro Institute for Neurological Research and Diagnostic, Cluj-Napoca, Romania; 2 Sociology Department, Babes-Bolyai University, Cluj-Napoca, Romania

**Interviewer: Stefana-Andrada Dobran**

**Interviewee: Professor Wayne Feng °**

° Professor of Neurology, Departments of Neurology, Stroke and Vascular Neurology, 2019.

Chief of Stroke & Vascular Neurology in the Departments of Neurology, Stroke and Vascular Neurology, 2019.

Professor of Biomedical Engineering, Department of Biomedical Engineering, 2022

Dr. Wayne Feng graduated in 1996 from the Medical University of South Carolina, where he completed his residency in neurology. He then received a stroke fellowship at Beth Israel Deaconess Medical Center/Harvard Medical School. He is board certified in general neurology as well as stroke neurology and specializes in young stroke diagnosis and management, stroke recovery, and rehabilitation. Additionally, Dr. Feng runs a first-of-its-kind clinic in South Carolina, the Post-Stroke Spasticity Clinic.

His research portfolios center on developing imaging biomarkers for stroke motor outcomes prediction and transcranial direct current stimulation (tDCS) to enhance post-stroke motor recovery. Dr. Feng has made several contributions to the field of brain stimulation, including demonstrating a dose-response relationship between current density and motor improvement through a meta-analysis. He further provided evidence that the electric field generated by tDCS in the human brain is dependent on the montage and dose used. Dr. Feng also showed that doubling the conventional tDCS dose from 2mA to 4mA is safe and tolerable to stroke patients.

Professor Feng has received multiple awards, including the stroke rehabilitation award from the International Stroke Conference 2015 and the Franz Gerstenbrand Award from the World Federation of Neurorehabilitation 2016. He has published numerous papers and has presented his research at regional, national, and international conferences. His philosophy on stroke care is "We will work together to get you better and stroke-free."

**S.A.D.:** Hello, dear Professor Feng! We are here, in Vienna, for the 12^th^ World Congress for NeuroRehabilitation, organised by the World Federation for NeuroRehabilitation. What is your first-hand opinion of the event, and have you participated in any previous editions?

**W.F.:** This is my fourth time attending a World Congress for NeuroRehabilitation, and this is the first one after Covid. Everything went so well, I was quite amazed to see so many attendees; everything went very well. I liked the General Assembly and the plenary sessions, and the e-poster sessions, the Special Interest Groups (SIGs) meeting; everything was just packed all together, and it went really well. I am very impressed.

**S.A.D.:** What is the overarching theme of this year’s congress, in your opinion?

**W.F.:** The main objective theme is to really get everybody connected after Covid, really foster neurorehabilitation in developing countries, to network all the professionals, and, in particular, foster young neurorehabilitation professionals.

**S.A.D.:** From your perspective, what is the role of hybrid multidisciplinary events in developing neurorehabilitation research and practice, and which similar avenues are worth exploring?

**W.F.:** I think this is a relatively new event; with Covid, we had to go with the hybrid, not just with online sessions and in-person sessions, and just meet everybody’s needs. Certainly, there were some challenges, particularly as it is at Christmas times, but it is a hybrid format, and everybody can participate; [and] really develop new strategies for neurorehabilitation. We need multidisciplinary teams; one person cannot do everything, so one really needs to foster interdisciplinary teams and teamwork efforts. I think this is going to be a new trend, and I expect it is going to do well.

**S.A.D.:** What are the advantages and limitations of transcranial direct current stimulation (tDCS)?

**W.F.:** That is an excellent question! I get a lot of questions like this. The advantages of a tDCS are really that it is simple, it is portable, the costs are relatively low, it’s very easy to use, it’s relatively safe, and it is a perfect tool for rehabilitation. Because we have other tools that seem fancier than tDCS, but you can’t import them, and they are hard to use and hard to translate for stroke patients. But tDSC has a lot of advantages, and it is low costs and can easily benefit a lot of patients. A limitation is really the fact that I think we need to do a little more research; it seems to me that the responses to the tDCS are quite a valuable instrument for the stroke patient, as well as for other patients with a neurological disease. For example, dosing – we are not yet very clear what is the optimal dose for a stroke patient. I think more research is required, we need to do more high-quality clinical trials to show the evidence so we can implement it in the clinical factors.

**S.A.D.:** How can tDCS be best used to avoid skin damage?

**W.F.:** Excelent question! In general, I think it is very safe for the brain and the skin. However, as I just pointed out, the dose is not well determined, and there are concerns that we might alter the dose for the brain, meaning we need an increase in the dose. If we increase the dose suddenly, we might cause some damage to the safety of the skin. Number 1 – I think we need to develop an electrodes technology to make sure that electrodes are much more friendly to the skin, second – we need [to do] a really good job to prepare the skin; for example, in my lab, we always suggest that you separate the hair very well, you put adequate saline on the electrodes, you leave the electrodes on the scalp for two or three minutes and just ensure the electrodes form a good contact with the scalp and then, you start the stimulation. Otherwise, it’s more likely to hurt the skin. But if you do those preparations, I think that the chances to have a skin injury are very, very low.

**S.A.D.:** What is the impact of mentoring programmes on the new generations of practitioners and researchers?

**W.F.:** I am the chair of the World Federation of NeuroRehabilitation Global Mentoring Programme. It is a new programme, but we certainly believe, together with the president and the members of the World Federation of NeuroRehabilitation, that we need to invest more in the young professionals because they are the future, not us; they are the future. To do this, we really invest in time and to connect senior mentors to a group of mentees and to share the success story of the mentor, share their experience with the mentee, and work with them, maybe one-on-one or one-on-two, in a very, very dedicated fashion, over a period of time. And really boost the career of those young professionals across the globe. In this way, we have a group of mentees that can quickly grow in their research and in their career and they can really continue to push neurorehabilitation to another level, not only in developing countries but also across the globe. I certainly believe this is something a professional society really should do in the long run!

**S.A.D.:** Thank you very much for your insight and for the interview!

**W.F.:** Thank you!

